# Non-Coding RNAs in Cancer Diagnosis and Therapy: Focus on Lung Cancer

**DOI:** 10.3390/cancers13061372

**Published:** 2021-03-18

**Authors:** Patricia Le, Giulia Romano, Patrick Nana-Sinkam, Mario Acunzo

**Affiliations:** Division of Pulmonary Diseases and Critical Care Medicine, Department of Internal Medicine, Virginia Commonwealth University, Richmond, VA 23298, USA; patricia.le@vcuhealth.org (P.L.); giulia.romano@vcuhealth.org (G.R.); patrick.nana-sinkam@vcuhealth.org (P.N.-S.)

**Keywords:** non-coding RNA, lung cancer, cancer diagnosis, cancer therapy

## Abstract

**Simple Summary:**

Researchers have spent nearly two decades unraveling the roles of non-coding RNAs in cancer biology. In recent years, deep transcriptomic profiling of tissue and circulating non-coding RNAs in cancer patients have elucidated non-coding RNAs as potential biomarkers that can inform cancer diagnosis and prognosis. Clinical trials have also begun examining non-coding RNA-based drugs as adjuncts to traditional chemotherapeutics. Overall, our review is structured to provide an overview of non-coding RNAs in cancer biology, diagnostics, and therapeutics, focusing on lung cancer.

**Abstract:**

Over the last several decades, clinical evaluation and treatment of lung cancers have largely improved with the classification of genetic drivers of the disease, such as EGFR, ALK, and ROS1. There are numerous regulatory factors that exert cellular control over key oncogenic pathways involved in lung cancers. In particular, non-coding RNAs (ncRNAs) have a diversity of regulatory roles in lung cancers such that they have been shown to be involved in inducing proliferation, suppressing apoptotic pathways, increasing metastatic potential of cancer cells, and acquiring drug resistance. The dysregulation of various ncRNAs in human cancers has prompted preclinical studies examining the therapeutic potential of restoring and/or inhibiting these ncRNAs. Furthermore, ncRNAs demonstrate tissue-specific expression in addition to high stability within biological fluids. This makes them excellent candidates as cancer biomarkers. This review aims to discuss the relevance of ncRNAs in cancer pathology, diagnosis, and therapy, with a focus on lung cancer.

## 1. Introduction

**Figure 1:**
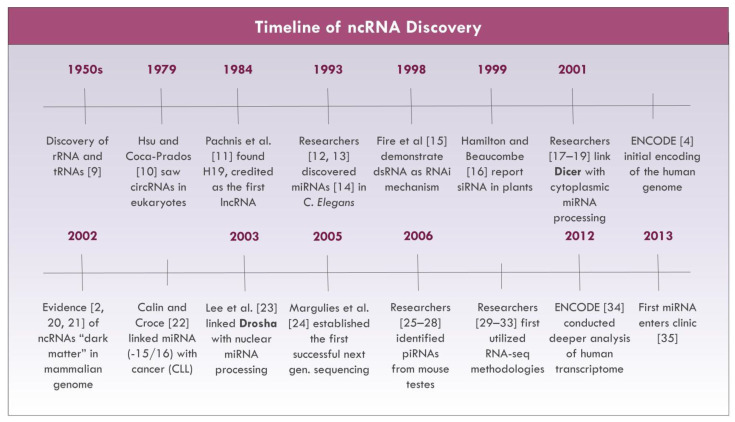
Non-coding RNA (ncRNA) research milestones [2,4,9,10,11,12,13,14,15,16,17,18,19,20,21,22,23,24,25,26,27,28,29,30,31,32,33,34,35].

The majority of the human transcriptome encodes for non-protein coding RNAs from intergenic, antisense, or overlapping regions to coded genes [1,2]. To date, it is estimated that non-coding RNAs (ncRNAs) account for approximately 98% of the human genome [3,4], with only a small fraction of this information having been studied thus far. Accordingly, investigations examining the “dark matter” [5] or non-coding transcriptome have risen exponentially within the last several decades (see Figure 1). Initially, these ncRNAs were believed to be “transcriptional noise”, [6] but recent advances in RNA sequencing technology and bioinformatics [7] have dispelled this notion by identifying the diverse contributions of ncRNAs on gene regulation. Although there are mixed opinions on the impact of ncRNAs on overall cellular activity [8], researchers remain optimistic about the importance of their roles in tumorigenesis and malignancy. In this paper, the role of ncRNAs in cancer pathophysiology, diagnostics, and therapeutics will be reviewed with an added focus on lung cancers.

## 2. Classifications of Non-Coding RNAs

Novel RNAs are discovered through various methodologies, including cDNA libraries, tiling arrays, and high-throughput sequencing technologies [36,37]. RNA sequencing (RNA-seq) technologies, in particular, have been invaluable in the elucidation of novel ncRNAs [38,39].

Classical housekeeping ncRNAs include ribosomal RNA (rRNA), transfer RNA (tRNA), small nuclear RNA (snRNA), and small nucleolar RNA (snoRNA) [40,41]. rRNAs and tRNAs have indispensable roles in protein synthesis: rRNAs are the primary RNA component of ribosomes, while tRNAs bring the correspondent amino acid per codon for building the primary peptide chain [41]. snRNAs are at the core of spliceosomes, which catalyze intron excision for mRNA maturation [41]. snoRNAs are involved in the post-transcriptional modifications and maturation of several RNA classes (e.g., rRNAs, snRNAs) [42].

The length of ncRNAs span from the magnitude of a few nucleotides to several thousands of nucleotides. Small non-coding RNAs (sncRNAs) are 20–30 nucleotides in length [43,44] and encompass microRNAs (miRNA), endogenous small-interfering RNA (endo-siRNA), and PIWI-interacting RNA (piRNA) [4,45,46,47]. These sncRNAs are involved with RNA interference (RNAi) whereby they form complexes with Argonaute family proteins to target and silence complementary mRNA transcripts [46]. Specifically, miRNAs and endo-siRNAs form RNA-induced silencing complexes (RISCs) with Argonaute proteins [46], while piRNAs interact with the PIWI clade of Argonaute proteins to form regulatory complexes [48]. Both miRNAs and siRNAs have been shown to target genes in a wide range of biological pathways and, as such, they have diversified functional consequences on cellular activity. The functions of piRNAs are more specific as they have been linked to regulating germline development and maintenance by preserving genome integrity [48].

Long non-coding RNAs (lncRNAs) have lengths greater than 200 nucleotides and include long intergenic non-coding RNAs (lincRNAs), long enhancer ncRNAs, and transcribed ultraconserved regions (T-UCRs) [4,36,45,49,50,51,52]. Other ncRNAs that are traditionally classified as lncRNAs include circular RNAs (circRNAs) [36], which are single-stranded closed loops [53] that range from 100 to 10,000 nucleotides in length [40], and pseudogenes [54,55], which are derived from previously coding transcripts. The functions of lncRNAs are more varied. In the nucleus, lncRNAs can regulate gene expression at both the transcriptional and epigenetic levels by altering transcriptional machinery activity and chromatin assembly, respectively [39]. In the cytoplasm, lncRNAs can regulate post-transcriptional mRNA processing, posttranslational protein modifications, and cell signaling pathways [39].

**Figure 2:**
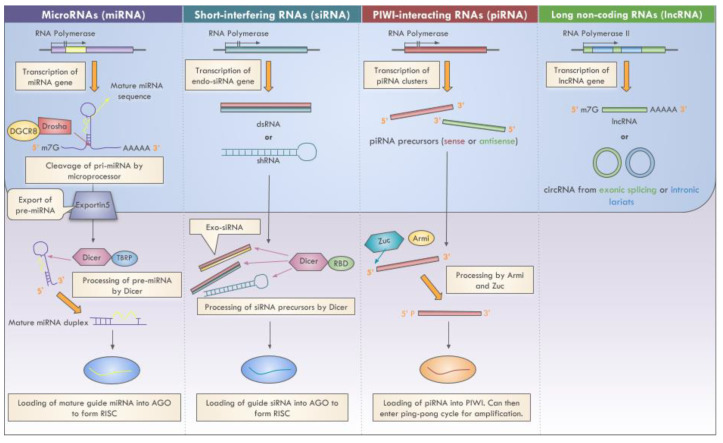
Biogenesis of select ncRNAs.

This review will mainly focus on select sncRNA and lncRNAs in cancer (Figure 2).

## 3. The Role of Non-Coding RNAs in Cancer Biology

In 2002, a pioneering study by Calin and Croce [22] identified a link between dysregulated miR-15a and miR-16-1 with chronic lymphocytic leukemia (CLL). Many ncRNAs have since been implicated in hallmark cancer processes [56,57]. Although only a few well-studied ncRNAs are discussed, it is worth noting that studying a single biomolecule or pathway in isolation simplifies the biological reality where many cancer pathways interplay and influence each other [40]. Furthermore, a single ncRNA can be involved in regulating multiple biological pathways [58], and can interact with a variety of DNA, mRNAs, proteins, and other ncRNAs [40,59,60]. This exemplifies the complexity of deducing the precise roles of ncRNAs in human cancer pathology.

### 3.1. Overview of ncRNAs in Cancer

With advances in RNA sequencing (RNA-seq) technologies, the transcriptome of cancer cells and tissues can be analyzed [38]. With this technology, the sequences and frequencies of dysregulated ncRNAs in cancers can be obtained [38,39].

Regarding the contributions of ncRNAs to human cancers, miRNAs are by far the most studied [61,62]. To uncover the cancer-related functions of miRNAs, numerous in vitro and in vivo experiments implement strategies of over- and under-expressing the miRNA(s) of interest. This is followed by examination of the induced biological activity through various functional assays [36,55]. Elucidating the mRNA targets of miRNAs through using in silico approaches (e.g., TargetScan, miRanda) or high-throughput sequencing also aids in the derivation of their function [36,63]. Interestingly, in conjunction with inducing RNAi, recent studies have shown that secreted miRNAs can also act as ligands for triggering prometastatic inflammatory responses within the tumor microenvironment [64,65]. The functions of piRNAs in cancer are less understood. Most studies thus far have examined the PIWI clade of Argonaute proteins independent of piRNAs, but more recent studies have examined the PIWI/piRNA interaction in cancers [48,66,67]. There is a general upregulation of these complexes in cancers, which has been linked to aggressive cancer phenotypes [48]. There are several well-established lncRNAs that have been linked to cancers (e.g., HOTAIR, H19, MEG3, MALAT1). They have varied roles in driving cancers and are specifically involved in post-transcriptional gene regulation, cell proliferation, metastasis, angiogenesis, and drug response. Overall, through the insight of functional studies, the effect of ncRNAs can be broadly categorized as being either tumorigenic or tumor suppressive, although some ncRNAs can demonstrate both activities in a context-dependent manner [68,69]. Some dysregulated ncRNAs are shown in Table 1.

Collectively, a variety of genetic and epigenetic factors can explain the deregulation of these ncRNAs in cancers, including gene amplification or deletion [22,62,120], transcriptional repression [121], abnormal biosynthesis [122,123,124], alternative splicing [125], and epitranscriptome modifications or “RNA editing” [59,126,127] (see [127] for review), with the latter encompassing nucleotide substitutions [127], methylation [128], and acetylation [129]. Furthermore, lncRNAs, especially circRNAs, can function as competitive endogenous RNAs (ceRNAs) against mRNAs for miRNA binding [53]. This prevents miRNAs from executing their regulatory functions [130]. For example, H19 has binding sites for let-7 that allow it to “sponge” or reduce let-7 availability for target mRNAs [130].

#### 3.1.1. miRNAs Dysregulated in Cancer

The first miRNAs connected to human cancers were miR-15a and miR-16-1. These miRNAs are commonly downregulated in CLL as a consequence of a chromosomal deletion localized at 13q14 [22]. Moreover, defective DROSHA processing can also reduce levels of miR-15/-16 [131]. Impaired miRNA processing and lowered expression of mature miRNAs are generally observed in cancers [122,123,124]. Functionally, miR-15a and miR-16-1 target B cell lymphoma 2 (Bcl2), an anti-apoptotic protein [94], and Cdc2 and Anxa2, which are cell cycle regulators [95]. Accordingly, the restoration of miR-15a and miR-16-1 significantly increases apoptosis in vitro [94], and reduces tumor size [95] and metastasis [96] in vivo. The miR-15/-16 cluster further mediates immunological response by negatively regulating T-cell differentiation, survival, and memory [132]. Since their initial connection to CLL, this cluster has been linked to other cancers as well [96,97,133,134]. Similarly, miR-29, another tumor suppressor, triggers senescence [135] and apoptosis in malignant cells. It targets Mcl-1, an anti-apoptotic protein of the Bcl family [98] that is commonly elevated in cancers [136,137,138]; DNMT3, a demethylase; and CDK6, a cell cycle regulator [100]. miR-29 is further associated with increased p53 activity [99]. A reduction in miR-29 expression is observed in a variety of human cancers [99,101,102,103]. Likewise, miR-34 has been linked with p53 activity [106,107,108,109]. Under normal conditions, physiological stress induces p53, which then acts as a transcriptional activator of numerous genes, including the miR-34 family [106]. The miR-34 family has been demonstrated to target cell cycle activators such as CDK4 and MET [106] along with anti-apoptotic factors such as Bcl2 [107]. Therefore, miR-34 is broadly associated with regulating numerous tumorigenic processes, including proliferation, apoptosis, epithelial-to-mesenchymal transition (EMT), invasiveness, differentiation, and metastasis [109,139]. Some additional highly characterized tumor suppressor miRNAs include the miR-200 family, which disrupts EMT by targeting E-cadherin transcriptional repressors such as ZEB1 [112,140], and the Let-7 family, which silences oncogenes such as RAS [141].

There are also several highly studied oncomiRs, including miR-21 [114,142,143,144], miR-155 [144,145], the miR-17-92 family [116,146,147], and miR-221/222 [118]. Mechanistically, these oncomiRs can function synergistically with other oncogenes (e.g., c-myc) [121], or they can directly target tumor suppressor genes (e.g., PTEN) [114,118] such as apoptotic factors (e.g., APAF1, CASP3) [114] or negative regulators of oncogenic pathways (e.g., SHIP1) [148]. Due to their marked contributions to hallmark cancer processes, some human malignancies demonstrate oncomiR addiction in which the cancer becomes reliant on the expression of specific oncogenic miRNA(s) for tumorigenesis [149].

#### 3.1.2. piRNAs Dysregulated in Cancer

The contributions of other sncRNAs, namely piRNAs, in cancer have been studied in more recent years [48,150]. Analogous to other sncRNAs, piRNAs form regulatory complexes with the PIWI clade of Argonaute proteins (e.g., PIWIL1, PIWIL2, PIWIL3, PIWIL4), and these complexes transcriptionally silence sequences with complementary genomic loci by [48]. In particular, the PIWI/piRNA pathway regulates germline development and maintenance by preserving genome integrity [48]. This is achieved through repressing the mobilization of transposable elements [48,151]. Characteristics of germ cells, such as rapid proliferation and continual self-renewal, are shared with cancer cells, prompting researchers to examine germline-specific factors in connection to cancer [151]. Early investigations focused on the contributions of dysregulated PIWI, rather than piRNAs, in cancers [151]. The consensus of the findings so far is that there is an upregulation of PIWI family members in cancers derived from both gonadal [66] and somatic cells [48,67,152]. PIWIL1 upregulation in pancreatic cancer has been physiologically linked to activating anaphase promoting complex (APC), which induces metastasis in a piRNA-independent fashion [152]. However, a similar study by Genzor and colleagues [153] reports that PIWIL1 upregulation in colon cancer cells results in a lack of functional PIWIL1/piRNA complexes with no observable piRNA-independent functions of PIWIL1. More recent efforts have begun examining the combined role of PIWI/piRNA complexes in cancers since these complexes may prevent mutations and subsequent genome instability due to transposon mobilization [48]. Upregulation of PIWI/piRNAs in human cancers have been frequently reported [48,66,67], where elevated PIWIs is associated with increased cancer aggressiveness [48]. These findings suggest that PIWI/piRNA silencing of transposon mobilization is not sufficient to explain their roles in cancer pathology [48]. Moreover, similar to miRNAs, aberrant expression of piRNAs, such as piR-651, piR-932, and piR-823, has been associated with various hallmark cancer processes, including tumorigenesis, angiogenesis, and metastasis [89,150,154,155]. A bioinformatic study by Martinez and colleagues [156] suggests that piRNAs demonstrate both pan-cancer and tumor-specific expression patterns, and that subgroup-specific piRNA expression can even delineate clinical features of specific tumors. Mechanistically, piRNAs have been shown to regulate gene expression by modulating epigenetic modifications of histone deacetylation and DNA methylation [154,157]. With novel methodologies and functional assays being established (e.g., multiplex bioimaging [158]), the contributions of piRNAs in cancers will be better understood.

#### 3.1.3. lncRNAs Dysregulated in Cancer

A large number of unique cancer-associated lncRNAs have also been identified [159]. Unlike sncRNAs, the mechanisms of lncRNAs in cancers can widely influence gene expression at the epigenetic, transcriptional, and post-transcriptional levels [55]. The Pan-Cancer Analysis of Whole Genomes (PCAWG) Consortium [159] recognizes the need for a resource detailing lncRNAs that have validated causative roles in cancer. This led to the Cancer LncRNA Census (CLC), a thorough compilation of 122 GENCODE-annotated lncRNA genes that have well-established cancer-related functions of which 77 are oncogenic, 35 are tumor suppressive, and 10 exhibit both activities [159]. The most widely observed lncRNAs in human cancers were found to be HOTAIR, a lncRNA encoded in the HOXC gene that interacts with Polycomb Repressive Complex 2 (PRC2), a histone methyltransferase, for methylating and silencing various tumor suppressor genes [70,72]; MALAT1, which is involved in alternative splicing [73,74]; MEG3, a tumor suppressor that regulates cell proliferation through p53 dependent and independent pathways [75]; and H19, which induces cell survival pathways in response to stressful conditions [77].

Pseudogenes and circRNAs are often classified as lncRNAs as well. Pseudogenes are derivatives of protein-coding genes that contain a defect which renders them non-coding [54,55,160]. Several pseudogenes have been implicated in cancers, including BRAFP1, which drives activation of BRAF and, more broadly, the MAPK pathway [78,160]; NANOG and OCT4, which sustain cell-renewal and pluripotency of embryonic stem cells [160]; and PTENP1, which sponges miRNAs that target PTEN, thereby increasing PTEN expression for suppressing tumorigenesis [55,81]. CircRNAs have been described to have numerous functions in tumor immunosurveillance, immune evasion, angiogenesis, cell permeability, cell death, and extracellular matrix (ECM) remodeling [161,162,163]. Although certain pro-tumorigenic circRNAs can be overexpressed in cancers, such as circPRKCI in lung adenocarcinoma [85] and circHIPK3 in colorectal cancer [87], global expression of circRNAs is generally decreased in cancers [164].

### 3.2. ncRNAs in Lung Cancer

Although lung cancer incidence has declined for both males and females over the last several decades [165], lung cancer remains the prevailing cause of cancer-related deaths [166,167]. Histological categorization of lung cancers is divided into small cell lung cancer (SCLC), which is characterized as being a highly malignant growth with neuroendocrine features and accounts for ~15% of cases, and non-small cell lung cancer (NSCLC), which accounts for the remaining ~85% of cases. The latter encompasses adenocarcinoma (LUAD) [168], squamous cell carcinoma (LUSC), and large cell carcinoma (LCC) [165].

One of the earliest lncRNAs connected to lung cancer is Metastasis Associated in Lung Adenocarcinoma Transcript (MALAT1) [169]. This lincRNA transcript was discovered as a prognostic marker of metastatic disease and overall survival in LUAD patients. MALAT1 has normal expression in various human tissues [169], and is important for normal physiological and developmental processes [170]. MALAT1 is primarily localized in nuclear speckles [171], and has been strongly associated with affecting alternative splicing [73,74]. To understand its role in lung cancer, Tano and colleagues [172] silenced MALAT1 in vitro and observed impaired cell motility due to a downregulation in genes involved with extracellular matrix and cytoskeleton rearrangement (e.g., HHMR, ROD1, CCT4, CTHRC1). MALAT1 knockdown had no observable effect on proliferation [172]. Gutschner and colleagues [173] conducted loss-of-function studies in which an antisense oligonucleotide inhibitor of MALAT1 was introduced to lung cancer xenograft mice models. Faulty cell migration and fewer lung tumor nodules in vivo were observed. Increased MALAT1 is also seen in NSCLC patients with brain metastasis compared to those without, providing further support for its role in lung cancer metastasis [174]. More broadly, MALAT1 upregulation has been observed in many human cancers [73,175], which suggests that it is a ubiquitous metastatic driver. Moreover, its role in cancer is likely context-dependent since MALAT1 is shown to have anti-metastatic effects in breast cancer [175].

Many aforementioned ncRNAs also contribute to the pathogenesis of lung cancer. Takamizawa and colleagues [176] were the first to identify a reduction in the let-7 family of miRNAs in lung cancers. When the researchers overexpressed let-7f in A549 LUAD cells, they observed a marginal reduction in colony formation. In a similar report from Kumar and colleagues [177], delivery of a lentivirus vector expressing let-7g into an autochthonous mice model of NSCLC was shown to suppress tumor initiation. However, formed tumors also expressed let-7g, potentially as a consequence of let-7g resistance. Mechanistically, let-7g acts on Ras [168] and HMGA2 [178] in lung cancers, which is consistent with findings of reduced Ras proteins and HMGA2 in let-7g expressing tumors [177]. MiR-34b/c are also downregulated in lung cancers [179,180,181]. Kim and colleagues [180] conducted RNA sequencing of miR-34b/c transduced 344SQ lung cancer cells and found that these miRNAs modulate cell adhesion genes. This can explain the reduction of anchorage-independent growth in 344SQ transduced with miR-34b/c. Low miR-34c is also seen in NSCLC-derived exosomes of which accelerates invasion and migration by upregulating integrin α2β1 [182]. Mizuno and colleagues [181] observed reduced cell migration, invasion, and proliferation when SCLC cells were transfected with miR-34. In their study, they used an in silico approach to identify four genes (TOP2A, MELK, CENPF, SOX1) as putative targets of miR-34b. TOP2A and MELK, in particular, appear important for the high proliferation and metastatic capabilities of SCLC [181]. Cortez and colleagues [183] also report that p53/miR-34 act synergistically to downregulate PDL1, an immunosuppressive protein, in NSCLC. In the lungs, miR-21 is the most abundant miRNA, and its expression is further elevated in lung cancers. It affects apoptosis, proliferation, angiogenesis, and survival through inhibiting known tumor suppressor genes such as PDCD4 and PTEN [114,184,185,186]. Frezzetti and colleagues [186] have also shown that activation of oncogenic Ras, which is observed in a population of patients with lung cancer, induces miR-21 expression during neoplastic transformation. This suggests that miR-21 contributes to early disease development.

Upregulation of lncRNA HOTAIR in lung cancers has been linked to lymph node metastasis [187,188,189]. Liu and colleagues [187] conducted several in vitro experiments in which they silenced or induced HOTAIR in lung cancer cell lines with high (SPC-A1, NCI-H1975) or low (A549) HOTAIR expression, respectively. They found that HOTAIR is associated with reduced apoptosis and increased migration. Furthermore, they found that repression of HOTAIR reduced metastatic nodules in vivo. They propose that HOTAIR promotes invasion and metastasis through deregulating EMT markers such as MMPs and HOXA5. LncRNA MEG3, on the other hand, is downregulated in NSCLC [190]. Lu and colleagues [190] observed that MEG3 induction markedly decreased cell growth and colony formation in vitro, and tumor growth and weight in vivo. The tumor-suppressive effects of MEG3 are associated with p53 activation. As mentioned, circPRKCI is amplified in LUAD [85]. Qiu and colleagues [85] report that circPRKCI promotes proliferation and migration by sponging miR-545 and miR-589, thus preventing them from inhibiting the pro-tumorigenic transcription factor E2F7.

Many ncRNAs are also involved in the acquisition of a treatment resistance phenotype. Several reports have shown that lung cancer cell lines with acquired resistance to standard chemotherapies (e.g., cisplatin, paclitaxel, gefinitib) have altered expression of hundreds of lncRNAs [191]. For instance, elevated HOTAIR and reduced MEG3 have been shown to drive cisplatin resistance in lung cancer cells [192]. In response to platinum-based chemotherapeutics (e.g., cisplatin), p53-stimulated ncRNAs (e.g., HOTAIR, PANDA) and DNA damage sensitive ncRNAs (e.g., lncRNA DDSR1) [193] can negate cell cycle arrest and elicit DNA repair, thereby overcoming drug-induced DNA damage in order to establish drug resistance [193]. Over-expression of lncRNA XIST induces autophagy, which is a protective mechanism engaged by cancer cells that ensures survival; as such, elevated XIST has been linked with poor cisplatin response [194]. Dong and colleagues [195] found that high GAS5 expressing cells (A549) exhibited increased cell death in vitro and reduced tumor formation in vivo especially when treated in combination with gefinitib, an epidermal growth factor receptor tyrosine kinase inhibitor (EGFR-TKI).

**Figure 3:**
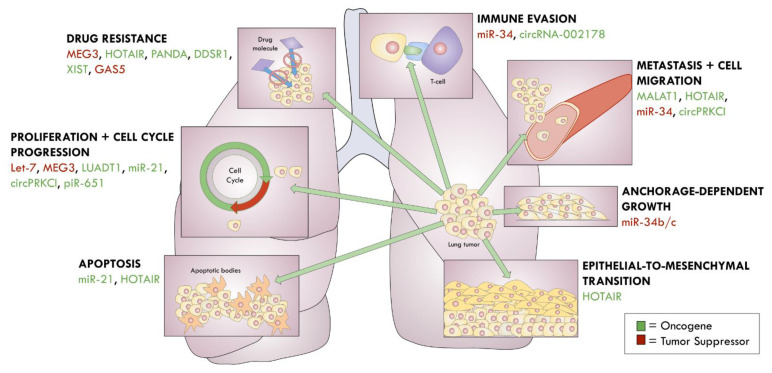
ncRNAs in lung cancer.

Novel ncRNAs are continuing to be linked to lung cancers. For example, Qiu and colleagues [196] recently identified lncRNA LUADT1, which is highly expressed in LUAD. LUADT1 associates with PRC2 and, together, they suppress p27 for inducing cell cycle progression. Wang and colleagues [197] also report that an upregulation of circRNA-002178 in LUAD acts to sponge miR-34, which consequently enhances PDL1 expression for immune evasion. Li and colleagues [89] found greater expression of piR-651 in a group of 78 NSCLC patients. When this piRNA is over-expressed in A549 cells, the researchers observed enhanced cell viability and metastasis, which they propose is mediated through the cyclin D1 and CDK4 pathways. Although the functional contributions of pseudogenes in lung cancer have not been extensively researched, Stewart and colleagues [198] recently found 104 pseudogene-derived lncRNAs to be dysregulated in LUAD, which prompts further investigation on their physiological relevance in lung cancer. A summary of the ncRNAs involved in lung cancers are shown in Figure 3.

## 4. The Use of Non-Coding RNAs as Cancer Biomarkers

Collectively, ncRNAs make excellent candidate biomarkers due to their high relative stability, unique expression profiles, and straightforward characterization by PCR [37]. Therefore, several clinical trials in the past decade have been conducted to identify ncRNA biomarkers in cancer patients for the purpose of developing screening tools. It is important for the intended application of the proposed biomarker(s) to be explicitly defined as predictive, prognostic, or diagnostic, as this will influence patient cohort composition and specimen selection [199,200].

Early studies of identifying tumor ncRNAs implemented the strategy of comparative profiling between normal and cancerous tissues [59]. Regarding clinical utility, tissue biopsies are well-established and informative, but invasive and not feasible for inaccessible tumors or vulnerable patients [201,202]. Furthermore, the information obtained from tissue biopsies is spatially and temporally dependent, thus it might provide an inaccurate representation of tumor heterogeneity and on-going tumor processes such as drug resistance [201,202,203,204]. Unique signatures of cancer-derived ncRNAs have been observed in circulation through biological fluids, including blood, saliva, and urine [205,206,207], leading researchers to explore the potential of liquid biopsies [202]. Compared to tissue biopsies, liquid biopsies are less invasive, which is optimal for screening [208] and treatment monitoring [202]. However, the frequency of circulating tumor cells (CTCs) is relatively low, and although ncRNAs can travel within bodily fluids independent of cells, free-travelling ncRNAs are prone to degradation by circulating RNAses [200,203]. An alternative approach is to examine ncRNAs encapsulated in extracellular vesicles (EVs) that are released by tumor cells [209]. Compared to normal cells, tumor cells have been shown to secrete a greater volume of vesicles for promoting cancer progression and pre-metastatic niche formation, thereby prompting investigations to more carefully assess their contents [200]. However, it is important to discern if altered levels of ncRNAs is confounded by differential vesicle quantities between cancer patients and healthy controls.

With the advent of high-throughput sequencing technologies such as next generation sequencing, whole genome expression profiles of patient and control samples can be compared to identify dysregulated ncRNAs [36,199]. Notably, it is unlikely that a single biomarker will be sufficient for disease characterization due to the heterogeneity of human cancers and ubiquitous expression of most ncRNAs [210,211]. Instead, bioclassifier systems composed of a panel of biomarkers may be necessary for achieving high sensitivity and specificity [210]. Large scale expression profiles of ncRNAs have already demonstrated capabilities in classifying poorly differentiated tumors, and can further contribute to our understanding of the dynamics underlying malignancies [123,200]. A major challenge with these bioclassifiers, however, is ensuring reproducibility. Thus, it is important to standardize sample processing and RNA extraction protocols, normalization methods, and bioinformatic analyses [199,207,211].

Although numerous promising ncRNA biomarker candidates have been identified, PCA3 is the only ncRNA to receive FDA approval as a biomarker thus far [55]. LncRNA PCA3 is uniquely upregulated in prostate cancer [212]. Hessels and colleagues [213] report an overexpression of PCA3 in urine obtained from patients with prostate cancer, thereby leading to the development of non-invasive PCA3 urine tests for clinical detection of early prostate cancer [214]. Presently, this test is being used in conjunction with other established tests (e.g., PSA blood test, TMPRSS2:ERG urine test) [55,215,216].

### 4.1. ncRNAs as Lung Cancer Biomarkers

The five-year survival rate for lung cancer (19%) ranks among the lowest for all cancers [167]. More than half of patients present with advanced-stage disease where curative therapies are limited [217,218]. Consequently, a sizable proportion of these patients are untreated [219]. Critically, lung cancer screening can improve the survival rates for high-risk patients. In screening programs, 80% of lung cancers are found at an early-stage; without screening, an overwhelming 70% of patients are diagnosed with late-stage disease [220]. Currently, the standard for lung cancer screening is a low-dose CT scan [162]. An increasing number of studies are showing that lung cancer biomarkers are promising adjuncts to CT scans for early disease detection and prognostic evaluation [221] (see Table 2).

#### 4.1.1. Disease and Diagnostic Biomarkers in Lung Cancer

In a study by Dou and colleagues [222], differential expression of plasma sncRNAs between early-stage LUAD patients and healthy controls were examined. To bypass challenges with normalization, the researchers employed a pair-based strategy in which they compared the ratios of ncRNA pairs within the same sample. The researchers found and validated a panel of seven small ncRNA pair ratios that was able to discern LUAD patients from healthy controls (AUC = 100% in the training cohort, 89.5% in the validation cohort). Dou and colleagues also generated a secondary panel of five ncRNA pairs to distinguish LUAD patients from patients with benign disease (AUC = 82% in the training cohort, 74.2% in the validation cohort). Similarly, Zaporozhchenko and colleagues [223] found a panel of ten plasma miRNA pairs that differentiated lung cancer patients from non-cancer controls (AUC = 97.9%). Lin and colleagues [224] examined plasma lncRNAs and found that a combination of SNHG1 and RMRP could distinguish NSCLC patients from controls with high sensitivity (84% = development cohort, 82% = validation cohort) and specificity (87.5% = development cohort, 86% = validation cohort). However, the researchers encountered challenges with reliably detecting the expression of ncRNAs by qRT-PCR, which exemplifies the difficulties associated with quantifying circulating ncRNAs.

Distinct ncRNA signatures coming from tumor-derived exosomes have been identified as well. Jin and colleagues [225] found that a combination of exosomal let-7b-5p, let-7e-5p, miR-24-5p, and miR-21-5p was able to distinguish stage-I NSCLC patients from healthy controls (AUC = 89.9%, sensitivity = 80.25%, specificity = 92.31%). Furthermore, they identified unique and shared exosomal miRNAs between LUAD and LUSC, where miR-181-5p and miR-361-5p were able to discern LUAD from other NSCLC patients (AUC = 93.6%, sensitivity = 80.65%, specificity = 91.67%), and miR-320b and miR-10b-5p were able to discern LUSC from other NSCLC patients (AUC = 91.1%, sensitivity = 83.33%, specificity = 90.32%). Similarly, Wang and colleagues [226] found that tissue circRNA-0001073 and circRNA-0001495 could differentiate LUAD and LUSC subtypes. Within the exosomes, Grimolizzi and colleagues [227] also found that an elevation of miR-126, a tumor suppressor, could distinguish early-stage NSCLC patients from controls. They hypothesized that tumor cells upregulate the secretion of tumor-suppressive miR-126 for removal through the exosomal pathway. When miR-126-containing exosomes derived from normal endothelial cells were forcibly endocytosed, targeted lung cancer cells displayed reduced growth and angiogenesis. A recent study by Best and colleagues [228] also found that RNA profiles, including ncRNAs from tumor-educated platelets could accurately detect early- and late-stage NSCLC. Specifically, through interacting with tumor cells, platelets sequester circulating tumor-associated biomolecules and, in response to external stimulation from the tumor environment, it can further undergo splicing of its pre-mRNA. The authors found that platelet RNA profiles are affected in nearly all cancers, including NSCLC, and that platelet RNA can supplement as an RNA onco-signature to tissue biomarkers such as KRAS mutations.

A-to-I editing of miRNAs is globally reduced in cancers [233]. Leveraging on this knowledge, edited miRNAs can serve as cancer biomarkers. Nigita and colleagues [229] analyzed edited miRNAs from small-RNA sequencing data of LUAD and LUSC tissues collected from the TCGA, and examined an independent cohort of plasma-derived exosomes from NSCLC patients. A significant downregulation of edited miR-411-5p was found in the exosomes of late-stage NSCLC patients compared to controls, which coordinated with reduced editing levels of miR-411-5p in NSCLC tissue samples from the TCGA. Maemura and colleagues [230] have also found significantly reduced miR-99a-5p editing from analyzing published small-RNA sequencing; this reduction was also observed in their cohort of 50 LUAD patient tissue samples.

#### 4.1.2. Prognostic and Treatment Biomarkers in Lung Cancer

In addition to being markers of early disease, ncRNAs can further inform prognosis, disease progression, and treatment response of lung cancer patients. In the same study by Maemura and colleagues, they had also found that decreased miR-99a-5p editing was associated with lowered overall survival in patients [230]. Asakura and colleagues [231] found that a combination of plasma miR-1268b and miR-6075 showed high sensitivity (95%) and specificity (99%) in predicting resectable lung cancer cases regardless of tumor histology and TNM staging. This study was particularly remarkable due to the large discovery (208 patients and healthy controls) and validation cohorts (1358 patients, 1970 healthy controls). Similarly, patients with decreased let-7 expression, independent of stage, have been observed to exhibit significantly worse prognosis after having undergone potentially curative tumor resection [176]. Notably, the prognostic value of let-7a for overall survival was found to be enhanced when examined in conjunction with elevated miR-155 [103]. Schmidt and colleagues [232] also found that positive expression of lncRNA MALAT1 in tumor tissue significantly correlated with poor prognosis for LUSC patients (*p* = 0.012).

## 5. The Use of Non-Coding RNAs as Cancer Therapeutics

As researchers unravel the diversity of RNAs, new strategies have been recognized for mimicking or antagonizing these nucleic acids [234]. Accordingly, the realm of RNA-based drugs has grown vastly and currently encompasses antisense oligonucleotides (ASOs), RNAi-based technologies (e.g., miRNAs and siRNAs), mRNA therapeutics, and single-guide RNAs (sgRNAs), with the latter being used to direct gene targeting of CRISPR-Cas9-based systems [234,235]. These therapeutic strategies can be designed to act on “non-druggable” targets where conventional therapeutics, which typically act at the protein-level, have previously failed [235,236].

Curating RNA medicine for disease treatment has primarily employed two strategies with respect to ncRNAs. One approach is to restore tumor suppressive ncRNAs. For miRNAs, this can be accomplished through the delivery of synthetic analogs called “mimetics” [237]. The first mimetic to enter clinical trials for cancer treatment was MRX34, an analog of the tumor suppressor miR-34, which is frequently reduced in human cancers [35,45]. MRX34 was delivered via a liposomal carrier to patients with advanced solid tumors [45,238]. Due to mixed treatment responses, along with several cases of adverse immunological reactions, the clinical trial has ceased for re-evaluation. It is possible that the delivery vehicle, rather than the drug, induced the immunological toxicity observed in some patients. Since MRX34, only one other mimetic has reached clinical trials: mesomiR-16, a miR-16 mimetic that is currently being evaluated for its efficacy in treating malignant pleural mesothelioma [239,240].

The second approach aims to silence genes implicated in disease pathogenesis. This can be accomplished by utilizing our knowledge of intrinsic RNAi pathways to engineer exogenous siRNAs and artificial miRNAs (amiRNAs) that transiently repress target gene expression at the mRNA level [241,242,243]. Several siRNA-based therapeutic agents have reached clinical trials for cancer treatment, including ALN-VSP02, a lipid nanoparticle-containing siRNAs against VEGF and KSP that is being tested on patients with advanced tumors who have at least one liver lesion [244]; Atu027, which is being used to treat advanced tumors and, more recently, pancreatic ductal cancer, through targeting PKN3 [245]; siG12D LODER, which delivers an siRNA against KRAS G12D for treating pancreatic cancer patients [246]; TKM-080301, which aims to reduce over-expressed PLK1 in patients with adrenocortical cancer [247]; and EPHARNA, a liposome-incorporated siRNA for EphA2 being used to treat patients with advanced solid tumors [248]. Furthermore, ASOs can be synthesized to target aberrantly expressed cancer-associated miRNAs and, to a lesser extent, lncRNAs. So-called “antagomirs” were demonstrated by Krützfeldt and colleagues [249] to be remarkably effective at silencing endogenous miRNAs, including miR-122. Currently, Miravirsen, a miR-122 antagonist, is being assessed for treatment of hepatitis C [250]. Regarding cancer treatment, Cobomarsen (MRG-106), an inhibitor of miR-155, is being evaluated for its ability to treat mycosis fungoides, a form of cutaneous T-cell lymphoma [251]. Current clinical trials implementing ncRNA-based therapeutics are in Table 3.

There are many aspects that are thoughtfully considered before these RNA drugs reach clinical trials. For example, to reduce the likelihood of off-target effects, designed molecules are subjected to in silico analyses (e.g., basic local alignment search tool (BLAST)) to evaluate its effect on gene expression [244]. Moreover, chemical modifications are often needed to increase the survivability of nucleic acids against circulating nucleases, and for negating immunological toxicity [244]. Such modifications include the addition of chemical groups at the 2′-position of ribose (e.g., 2′-O-methyl, 2′-O-methoxyethyl) and the substitution of nucleotides for locked nucleic acids [243,244,252]. A report by Cheng and colleagues [253] further showed that attachment of antagomirs to a peptide with a low pH-induced transmembrane structure (pHLIP) can support the molecule within acidic tumor environments. Interestingly, Li and colleagues [254] found that delivery of siRNAs preassembled with Ago2 showed more potent and sustained gene silencing. However, cancer cells have been shown to evade delivered miRNA therapeutics by secreting the miRNAs complexed with Ago2 [255].

The selection of delivery method is also imperative for ensuring that the drug reaches its intended target rather than be eliminated via renal clearance [244]. Drug delivery can be non-targeted (e.g., nanoparticles) or targeted (e.g., conjugated to antibodies, ligands, aptamers) [243]. Furthermore, the drugs can be delivered regionally or systemically. Through either route, the molecules must overcome barriers of extravasation, transport within the interstitium, cellular internalization, and localization to appropriate cellular compartment for action [256]. Altogether, RNA medicine is an incredibly exciting development but, as with any drug design, it must circumvent challenges in toxicity, clearance, survivability, targeting, and cellular uptake [257].

### ncRNAs for the Treatment of Lung Cancer

The benefits of implementing ncRNAs for lung cancer therapy have been assessed widely in preclinical models. Compared to siRNAs, miRNAs display imperfect complementarity to mRNAs such that it has the unique ability to affect multiple genes with fewer molecules required [242,258]. Acunzo and colleagues [243] developed amiRNAs for selectively targeting point mutations in KRAS deemed “non-druggable” in NSCLC. Taking advantage of the unique nature of the seed sequence of miRNAs, the researchers synthesized amiRNAs that demonstrate perfect binding to mutant KRAS G12S yet imperfect matching to KRAS WT. Compared with traditional siRNA-based drugs, the amiRNA-KD2 was able to discriminate KRAS G12S and WT lung cancer cells while retaining the ability to exert favorable effects on proliferation, migration, and necrosis. Furthermore, this strategy can be utilized to target other point mutations observed in cancer. The advantage of this type of drug design is that it minimizes toxicity against normal cells.

Vast studies have examined the effect of restoring tumor suppressor ncRNAs in preclinical lung cancer models. For instance, several groups have observed desirable effects from restoring miR-34 in reducing tumor burden in murine models of NSCLC [259,260,261], with similar efforts having been made to examine the effects of restoring other tumor suppressors such as miR-29 [262], let-7 [177], and MEG3 [190]. Some ncRNAs have been targeted in lung cancer treatment studies as well, including miR-21 [186] and MALAT1 [173]. Importantly, a variety of targeted delivery strategies have been rigorously evaluated by these research groups. Wu and colleagues [100] report success using cationic lipoplexes to deliver miR-29b to xenograft mice. Comparatively, Perepelyuk and colleagues [252] synthesized a hybridized nanoparticle with a MUC1-aptamer for targeting delivery of miR-29b to NSCLC cells expressing MUC1, a transmembrane protein that is aberrantly expressed by cancer cells. More broadly, new methodologies and systems are constantly being developed to study lung cancer and deliver therapeutic molecules. In a recent publication by Jeong and colleagues [263], they used human-cell derived exosomes for delivering miR-497 in vitro. In their study, the researchers created a model whereby A549 NSCLC cells were co-cultured with normal human endothelial cells (HUVEC) in a microfluidic device to better replicate the 3D tumor microenvironment.

As new discoveries within RNA medicine are made, pharmaceutical companies are continuing to lead clinical explorations and developments of RNA-based drugs [264]. Although no ncRNA-based drugs have been specifically curated for treating lung cancers, some of the aforementioned drugs have been utilized for treating advanced solid tumors including lung cancers. Furthermore, once the efficacy of varying RNA-based drugs in preclinical lung cancer models is determined, more RNA-based drugs can reach clinical trials.

## 6. Conclusions

In the last several decades, the field of ncRNA research has experienced a noticeable surge in discoveries and innovations. There are numerous advantages to utilizing ncRNAs as biomarkers, including their relative stability and straightforward characterization by PCR. Traditionally, clinical trials for identifying cancer biomarkers aim to build panels of clinically informative ncRNAs without screening for more nuanced editing at the post-transcriptional level. By diversifying the screening process to encompass modified ncRNAs, this can help to strengthen the sensitivity and specificity of proposed biomarker panels. In addition to being potential biomarkers, these dysregulated ncRNAs can have functionally relevant roles in malignancies. For instance, several ncRNAs, such as MALAT1, miR-34, and the let7 family, have now been robustly linked to inducing cellular pathways that maintain the malignant phenotypes of lung cancers.

Furthermore, understanding the degree to which these ncRNAs are dysregulated have helped researchers to develop RNA-based therapeutics, such as mesomiR-16 and Cobomarsen, which aim to restore or suppress aberrantly expressed ncRNAs. Additionally, by leveraging on the RNAi activity of sncRNAs, synthetic siRNAs and amiRNAs can be utilized to target aberrantly mutated genes, such as KRAS, which are commonly observed in lung cancers. However, the inherent obstacles of achieving reproducibility, sensitivity, and specificity; building representative in vivo systems; tumor heterogeneity; biomarker normalization and protocol standardization; drug delivery; and drug-resistance pathways have generated challenges in translating these molecules from the bench to the bedside. In lung cancer, resistance to platinum-based chemotherapeutics can, in part, be explained through modulation of DNA repair pathways and cell cycle arrest by lncRNAs such as HOTAIR and MEG3. Overall, due to discussed challenges, potential RNA-based therapies curated for lung cancers have been mainly examined in preclinical mice models. However, although the challenges in quantification, detection, and delivery of ncRNAs have strained the rate at which these molecules can achieve clinical success, this field is still relatively young and has already deepened our understanding of lung cancer biology.

## Figures and Tables

**Table 1:** Dysregulated ncRNAs in cancer.

Variable	ncRNA	Mechanism	Dysregulated in	Functions in Cancers	Ref.
lncRNA	HOTAIR	Oncogene	Endometrial, lung, ovarian, prostate, thyroid	Interacts with PRC2 to methylate and silence tumor suppressor genes	[70,71,72]
	MALAT1	Oncogene + tumor suppressor	Breast, endometrial, lung, ovarian, prostate, thyroid	Alternative splicing, metastasis	[71,73,74]
	MEG3	Tumor suppressor	Breast, colorectal, gastric, liver, lung, ovarian, prostate	Regulates proliferation, angiogenesis, epithelial-to-mesenchymal transition, drug sensitivity	[71,75,76]
	H19	Oncogene + tumor suppressor	Bladder, breast, colorectal, endometrial, ovarian, prostate	Induces cell survival pathways in response to stress, epithelial-to-mesenchymal transition (primary site) and mesenchymal-to-epithelial transition (secondary site)	[77]
	BRAFP1	Oncogene	Lymphoma	Activates BRAF	[78]
	NANOG	Oncogene	Breast, colorectal, hepatocellular, leukemia, lung, pancreatic, prostate	Sustains cell-renewal and confers stem cell-like properties. Involved with proliferation, migration, invasion, drug resistance	[79]
	OCT4	Oncogene	Liver, lung, pancreas	Sustains cell-renewal and confers stem cell-like properties. Involved with proliferation, migration, invasion, drug resistance	[80]
	PTENP1	Tumor suppressor	Breast, gastric, prostate, renal	Sponges microRNAs that target PTEN	[55,71,81,82,83,84]
	circPRKCI	Oncogene	Glioma, lung	Promotes proliferation and migration by sponging tumor suppressing miRNAs (e.g., miR-545)	[85,86]
	circHIPK3	Oncogene	Breast, colorectal, gallbladder, gastric, ovarian	Promotes cancer growth and metastasis by sponging tumor suppressing miRNAs (e.g., miR-7, miR-193a)	[87,88]
piRNA	piR-651	Oncogene	Lung	Enhances cell viability and metastasis	[89]
	piR-823	Oncogene + tumor suppressor	Colorectal, esophageal, gastric	Affects cell growth, metastasis, DNA methylation, apoptosis, transcriptional activity	[90,91,92]
	piR-932	Oncogene	Breast	Epithelial-to-mesenchymal transition	[93]
miRNA	miR-15/16	Tumor suppressor	Chronic lymphocytic leukemia (CLL), prostate cancer, colorectal, pleural mesothelioma	Enhances apoptosis, reduces tumor size and metastasis, and regulates immunological response	[94,95,96,97]
	miR-29	Tumor suppressor	Breast, head and neck, pancreatic, prostate, liver, lung, pancreas	Induces senescence and apoptosis. Mitigates against cancer metabolism proliferation, migration, and invasion	[98,99,100,101,102,103,104,105]
	miR-34	Tumor suppressor	Breast, glioblastoma, lung, pancreatic, prostate	Activated by p53. Affects proliferation, apoptosis, differentiation, epithelial-to-mesenchymal transition, invasiveness, and metastasis	[71,106,107,108,109,110,111]
	miR-200 family	Tumor suppressor	Breast, ovarian, lung, pancreatic, prostate, renal	Disrupts epithelial-to-mesenchymal transition, invasiveness, differentiation, and metastasis	[11,71,104,110]
	let7	Tumor suppressor	Breast, colorectal, gastric, liver, lung, renal, thyroid	Silences oncogenes (e.g., RAS), decreases stemness of cancer cells, regulates cell cycle and signaling pathways, inhibits epithelial-to-mesenchymal transition	[110,112,113]
	miR-21	Oncogene	Brain, breast, endometrial, liver, lung, pancreatic, prostate, thyroid	Induces cell proliferation pathways, metastasis, and regulates apoptosis	[71,104,114]
	miR-155	Oncogene	Breast, cervical, colorectal, leukemia, liver, lung, pancreatic, thyroid	Implicated in promoting cell survival and proliferation, anti-apoptosis, metabolic shift (Warburg effect)	[104,110,115]
	miR-17–92 cluster	Oncogene	Breast, colorectal, head and neck, leukemia, lung, lymphoma, pancreatic, renal	Functions with C-MYC and transcriptional regulation more broadly. Regulates proliferation, apoptosis, angiogenesis	[110,116,117]
	miR-221/222	Oncogene + tumor suppressor	Breast, endometrial, glioblastoma, hepatocellular, pancreatic, prostate, thyroid	Targets tumor suppressors. Induces cell proliferation, drug resistance	[104,118,119]

**Table 2:** ncRNA biomarkers in lung cancer.

	Biomarker	Sample	Clinical Information	Ref.
Disease/ Diagnostic	Seven paired miRNA panel	Plasma	Distinguishes early-stage LUAD + benign disease from control	[222]
	Five paired miRNA panel	Plasma	Distinguishes early-stage LUAD from benign disease	[222]
	Ten paired miRNA panel	Plasma	Distinguishes NSCLC (LUAD + LUSC) from controls (healthy/endobronchitis patients)	[223]
	SNHG1 + RMRP	Plasma	Distinguishes NSCLC from cancer-free controls	[224]
	Let-7b-5p, let-7e-5p, miR-24-5p, and miR-21-5p	Exosome	Distinguishes stage-I NSCLC patients from healthy controls	[225]
	miR-181-5p + miR-361-5p	Exosome	Can discern LUAD from other NSCLC histologies	[225]
	miR-320b + miR-10b-5p	Exosome	Can discern LUSC from other NSCLC histologies	[225]
	circRNA-0001073 + circRNA-0001495	Tissue	Differentiate LUAD and LUSC	[226]
	miR-126	Exosome	Distinguishes early-stage NSCLC patients from healthy controls	[227]
	RNA panels including ncRNAs	Tumor-educated platelets	Early- and late-stage NSCLC detection	[228]
	Edited miR-411-5p	Tissue + Exosomes	Distinguishes late-stage NSCLC from controls	[229]
	Edited miR-99a-5p	Tissue	Distinguishes LUAD from controls	[230]
Prognostic/ Treatment	Edited miR-99a-5p	Tissue	Informs of shorter overall survival and recurrence-free survival after resection of LUAD	[230]
	miR-17-3p miR-1268b + miR-6075	Plasma	Predicts resectable lung cancers regardless of histology and staging	[231]
	Let-7	Tissue	Informs of poor prognosis following tumor resection	[176]
	Let-7a + miR-155	Tissue	Informs of overall survival	[103]
	MALAT1	Tissue	Informs of poor prognosis for LUSC patients	[232]

**Table 3:** Current clinical trials with ncRNA-based therapeutics.

Clinical Trial ID	Stage	Disease	Therapeutic
NCT03608631	Phase I	Pancreatic cancer	**iExosomes** derived mesenchymal stromal cells with KRAS G12D siRNA
NCT01591356	Phase I	Advanced or recurrent solid tumors	**EphA2-targeting** DOPC-encapsulated siRNA
NCT00938574	Phase I	Advanced solid tumors	**Atu027** (siRNA targeting PKN3)
NCT00882180 + NCT01158079	Phase I	Advanced solid tumors with liver involvement	**ALN-VSP02** (lipid nanoparticle with siRNA targeting VEGF-A + KSP)
NCT03087591 + NCT02166255	Phase I	Metastatic solid neoplasms	**APN401** (peripheral blood mononuclear cells transfected with cbl-b siRNA)
NCT02369198	Phase I	Pleural mesothelioma, NSCLC	**TargomiRs** (miR-16 mimic)
NCT00689065	Phase I (terminated)	Solid tumors	**CALAA-01** (siRNA targeting RRM2)
NCT01829971 + NCT02862145	Phase I (terminated)	Solid tumors, liver cancer	**MRX34** (miR-34 mimic)
NCT01188785 + NCT01676259	Phase I + II	Pancreatic cancer	**siG12D-LODER** (siRNA targeting KRAS G12D)
NCT01437007	Phase I/II	Primary/secondary liver cancer	**TKM-080301** (siRNA targeting PLK1)
NCT02110563 + NCT02314052	Phase I/II (terminated)	Solid tumor, multiple myeloma, lymphoma	**DCR-MYC** (siRNA targeting MYC)
NCT03713320 + NCT03837457	Phase II (terminated)	Cutaneous T-Cell Lymphoma/Mycosis Fungoides	**Cobomarsen/MRG-106** (oligonucleotide inhibitor of miR-155)
